# Multiple myeloma cells expressing low levels of CD138 have an immature phenotype and reduced sensitivity to lenalidomide

**DOI:** 10.3892/ijo.2012.1545

**Published:** 2012-07-04

**Authors:** YAWARA KAWANO, SHIHO FUJIWARA, NAOKO WADA, MIKIKO IZAKI, HIROMICHI YUKI, YUTAKA OKUNO, KENICHI IYAMA, HIROSHI YAMASAKI, AKIRA SAKAI, HIROAKI MITSUYA, HIROYUKI HATA

**Affiliations:** 1 Department of Hematology, Kumamoto University School of Medicine;; 2 Department of Surgical Pathology, Kumamoto University Hospital;; 3 Department of Hematology and Oncology, Kumamoto City Hospital, Kumamoto;; 4 Department of Radiation Life Sciences, Fukushima Medical University School of Medicine, Fukushima;; 5 Department of Hematology, Faculty of Life Sciences, Graduate School of Health Sciences, Kumamoto University, Kumamoto, Japan

**Keywords:** multiple myeloma, CD138, IRF4, lenalidomide

## Abstract

CD138 expression is a hallmark of plasma cells and multiple myeloma cells. However, decreased expression of CD138 is frequently observed in plasma cells of myeloma patients, although the clinical significance of this is unclear. To evaluate the significance of low expression of CD138 in MM, we examined the phenotypes of MM cells expressing low levels of CD138. Flow cytometric analysis of primary MM cells revealed a significant decrease in CD138 expression in patients with relapsed/progressive disease compared with untreated MM patients. Patients with low levels of CD138 had a worse overall survival compared with patients with high levels of CD138, in newly diagnosed patients and patients receiving high-dose chemotherapy followed by autologous stem-cell transplantation. Two MM cell lines, KYMM-1 (CD138^−^ low) and KYMM-2 (CD138^−^ high), were established from a single MM patient with decreased CD138 expression. High expression of *BCL6* and *PAX5*, and downregulation of *IRF4*, *PRDM1* and *XBP1* was observed in KYMM-1 compared with KYMM-2 cells, indicative of the immature phenotype of KYMM-1. KYMM-1 was less sensitive to lenalidomide than KYMM-2, while no difference in sensitivity to bortezomib was observed. KYMM-2 cells were further divided in CD138^+^ and CD138^−^ fractions using anti-CD138-coated magnetic beads. CD138^−^ cells sorted from the KYMM-2 cell line also showed high BCL6, low IRF4 expression and decreased sensitivity to lenalidomide compared with CD138^+^ cells. Our observations suggest that low CD138 expression relates to i) poor prognosis, ii) immature phenotype and iii) low sensitivity to lenalidomide. The observed distinct characteristics of CD138 low MM cells, suggest this should be recognized as a new clinical entity. Establishment of a treatment strategy for MM cells expressing low levels of CD138 is needed to improve their poor outcome.

## Introduction

Multiple myeloma (MM) is a B-cell neoplasia, characterized by the clonal expansion of malignant plasma cells in the bone marrow. During normal B-cell development, cells acquire expression of CD138, also known as syndecan-1 (SDC1), a marker highly specific for terminally differentiated normal plasma cells (1). CD138 is a heparin sulphate proteoglycan that controls tumor cell survival, growth, adhesion and bone cell differentiation in MM (2). Since CD138 is a specific surface antigen for MM cells and plasma cells in the bone marrow (3), it has been used for the purification of MM cells from clinical samples (4) and in the classification of MM cells in gene expression profiling analyses (5). In addition, the use of specific antibodies targeting CD138 are also considered as a novel treatment strategy for MM (6).

However, several studies have also reported decreased expression of CD138 in MM (7,8). Matsui *et al*, reported the existence of highly clonogenic MM cells lacking CD138 expression, and suggested that these cells may represent MM ‘stem cells’ (9). Furthermore, a decrease in CD138 expression has been observed during the course of clinical treatment in some patients. While high expression of CD138 is not consistently observed in myeloma cells, the significance of decreased CD138 expression in MM cells remains unclear.

In the present study, we investigated the expression of CD138 in primary MM cells by flow cytometry using the CD38 gating method (10), and analyzed the association between CD138 decrease and patient survival, retrospectively. To investigate the effects of decreased CD138 in MM, we utilized two MM cell lines with heterogeneous expression of CD138 (KYMM-1, CD138 low and KYMM-2, CD138 high). These cell lines were simultaneously established from the pleural effusion of an MM patient, who displayed a gradual reduction in CD138 expression during disease progression.

## Materials and methods

### 

#### MM patients and flow cytometric analysis

Flow cytometry data were obtained from 90 newly diagnosed and 15 relapsed or progressive MM patients, admitted to Kumamoto University Hospital, Kumamoto City Hospital and Hiroshima University Hospital. Written, informed consent was obtained according to the Declaration of Helsinki. Flow cytometry was performed by the commercially available CD38 multi-analysis (SRL Laboratories, Tokyo, Japan) by gating CD38 strong positive (++) fractions as previously described (10). To rule out the possibility of B-cell contamination, patients with CD19 positive cells and without cytoplasmic light chain restriction, were excluded from the analysis. Patients with greater than 20% CD138 negative cells in CD38^++^ fraction were termed ‘CD138 low’ patients, while patients with less than 20% of CD138 negative cells were termed ‘CD138 high’. The characteristics of the patients are summarized in Table I.

### Establishment of two MM cell lines from a single MM patient

#### Case report

A 75-year-old Japanese female was admitted to hospital with lumbago. Magnetic resonance imaging (MRI) examination revealed a plasmacytoma in the lumbar vertebra, accompanying lumbar fractures. Laboratory tests indicated the presence of the IgG-k type M-protein in the serum (IgG 3,849 mg/dl). Pathological examination of bone marrow aspirates revealed that 30.8% of the cells were plasma cells and she was diagnosed with symptomatic myeloma. A treatment regimen including melphalan, prednisolone and thalidomide therapy, combined with radiation against the plasmacytoma was initiated. Although transient regression of the disease was obtained, enlargement of the plasmacytoma was observed and pleural effusion contained almost 100% of plasma cells. Despite treatment with lenalidomide, the patient died 16 months after admission.

#### Establishment of cell lines

Mononuclear cells were separated from the pleural effusion fluid by Ficoll-Paque Plus (GE Healthcare, Uppsala, Sweden). Separated cells were collected and divided in 12-well plates and cultured in RPMI-1640 medium containing 10% fetal bovine serum at 37°C under 5% CO_2_. Media were replaced every 3 days. Two cell lines, referred to as KYMM-1 and KYMM-2, were obtained from different wells. These cells had been maintained for more than 450 days in culture and their viability was retained following repeated freeze/thaw cycles. The clonality of these two cell lines and primary samples was confirmed by Southern blot analysis using a DNA probe recognizing the immunoglobulin heavy-chain joining (Ig-JH) region (SRL Laboratories). Genomic DNA obtained from the patient’s pleural effusion fluid, KYMM-1 and KYMM-2 cell lines, was further analyzed using the Cell ID™ System (Promega, Madison, WI, USA), which identifies 9 short tandem repeat (STR)-loci and Amelogenin, to confirm the origin of cell lines. Epstein-Barr (EB) virus genome detection was performed by PCR using BamW region primers (SRL Laboratories) as previously reported (11).

#### Cell lines and cell culture

Human myeloma cell lines, KMM-1 (12), KMS-11 (13), KMS-12-BM (14), KMS-12-PE (14), U266 (15), KYMM-1, KYMM-2 and the human leukemia cell line, HL-60 (16), were cultured in RPMI-1640 medium containing 10% fetal bovine serum at 37°C under 5% CO_2_. KMM-1, KMS-12-BM and KMS-12-PE were kindly provided by Dr Ohtsuki (Kawasaki Medical School, Kurashiki, Japan).

#### Flow cytometric analysis of MM cell lines

MM cell lines were stained with fluorescent-labeled antibodies, PE-CD19 (clone HIB19), PE-CD20 (clone L27), FITC-CD38 (clone HIT2), PE-CD44 (clone 515), PE-CD45 (clone HI30), PE-CD138 (clone MI15) (BD Biosciences, Franklin Lakes, NJ, USA), PE-CD27 (clone O323), PE-CD54 (clone HCD54), FITC-CD56 (clone HCD56) (Biolegend, San Diego, CA, USA), PE-CD34 (clone 581) (Beckman Coulter, Brea, CA, USA) and FITC-κ light chain (Dako, Glostrup, Denmark). Flow cytometric analysis was performed using an EPICS MCL/XL flow cytometer (Beckman Coulter).

#### cDNA synthesis and reverse transcription-polymerase chain reaction (RT-PCR)

RNA was extracted from purified myeloma cells using TRIzol reagent (Invitrogen, Carlsbad, CA, USA). cDNA synthesis was performed using the SuperScript III First-Strand Synthesis System for RT-PCR (Invitrogen) according to the manufacturer’s protocol.

The expression of *SDC1* (*CD138*), *BCL6* and *PAX5* was determined by RT-PCR. *GAPDH* was used as a normalization control. Primers for *PAX5*(17) and *GAPDH*(18) were previously described. Primers for *SDC1* and *BCL6* were as follows: SDC1 (forward 5′-GCCGCAAATTGTGGCTACT-3′, reverse 5′-GCTGCGTGTCCTTCCAAGT-3′), BCL6 (forward 5′-GAG AAGCCCTATCCCTGTGA-3′, reverse 5′-TGCACCTTGGTGTTGGTGAT-3′).

Quantitative real-time RT-PCR was performed using Assay-on-Demand primers and Taqman Universal PCR Master mix reagent (Applied Biosystems, Foster City, NJ, USA). Samples were analyzed using the ECO™ Real-Time PCR System (Illumina, San Diego, CA, USA). The ΔΔCt method was utilized to analyze the relative changes in gene expression as previously described (19) using *β-actin* (*ACTB*) as a normalization control. The following primers and probes were used: *SDC1* (Hs00896423_m1), *IRF4* (Hs01056534_m1), *PRDM1* (Hs00153357_m1), *XBP1* (Hs00964360_m1), *BCL6* (Hs00277037_m1) and *ACTB* (Hs99999903_m1).

#### Detection of methylation

DNA methylation was analyzed by bisulfite sequencing. CpG islands spanning the transcription initiation site of the *SDC1* gene were identified by Methyl Primer Express v1.0 software (Applied Biosystems). A 362 bp DNA fragment of the region of *SDC1* containing CpG islands was amplified using the following primers: forward 5′-AGTATTTTGTGGAGTGTAGGAAGAA-3′, reverse 5′-CCTTTCAACTCRACTACTCCCT-3′. Genomic DNA was treated with sodium bisulfite as previously described (20) and subjected to 35 cycles of PCR. PCR products were directly sequenced for evaluation of methylation status.

#### Cell viability assay and detection of apoptosis

Cell viability was determined by WST-8 assay using the Cell Counting Kit-8 (Dojindo, Kumamoto, Japan). Briefly, cells were seeded in 96-well plates and treated with bortezomib (Janssen Pharmaceutical, Tokyo, Japan) or lenalidomide (Santa Cruz Biotechnology, Santa Cruz, CA, USA) for 24 or 72 h, respectively. Following treatment with each compound, cells were incubated with WST-8 reagent for 5 h. The absorbance of each well was measured at 450 nm using a VMax absorbance microplate reader (Molecular Devices, Sunnyvale, CA, USA). Apoptosis and cell death were evaluated using the Annexin V-FITC Apoptosis Detection Kit (MBL, Nagoya, Japan), according to the manufacturer’s instructions.

#### Western blot analysis

Antibodies against IRF4 (clone M-17) and actin (clone C-2) were purchased from Santa Cruz Biotechnology. Cell lysates were prepared using M-PER mammalian protein extraction reagent (Pierce Biotechnology Inc., Rockford, IL, USA) after addition of Halt EDTA-free phosphatase inhibitor cocktail and Halt protease inhibitor cocktail (Pierce Biotechnology Inc.). Cell lysates were separated in NuPAGE Bis-Tris precast gels (Invitrogen) and transferred to PVDF membranes using an iBlot Dry Blotting system (Invitrogen). Membranes were blocked with 5% non-fat dry milk for 1 h at room temperature, followed by incubation with a primary antibody at 4°C for 12 h. Membranes were then incubated with horseradish peroxidase conjugated rabbit anti-goat (Bethyl Laboratories, Inc., Montgomery, TX, USA) or sheep anti-mouse secondary antibodies (GE Healthcare, Little Chalfont, UK) for 1 h at room temperature. Antibody-bound proteins were visualized using ECL prime western blotting detection reagent (GE Healthcare) and a bio-image analyzer LAS-1000 (GE Healthcare). The density ratio of the protein bands was calculated using Image J software (National Institutes of Health, Bethesda, MD, USA).

#### Immunohistochemistry

Immunohistochemistry was performed on paraffin-embedded bone marrow aspirated tissue sections, using anti-CD138 (clone MI15, Dako) and anti-IRF4 (clone MUM1p, Dako) antibodies, according to the manufacturer’s instructions.

#### CD138 magnetic cell sorting

CD138^+^ and CD138^−^ fractions of KYMM-2 cells were separated using CD138-immunomagnetic beads (Miltenyi Biotech, Paris, France) according to the manufacturer’s protocol. The magnetic cell sorting was conducted twice to increase the purity of each fraction. The purity of each fraction was determined as approximately 90%, by flow cytometry.

#### Statistical analysis

The number of CD138^−^ cells in the CD38^++^ fraction was compared using the Mann-Whitney U test. Patient survival was calculated by the Kaplan-Meier method. For comparisons of survival curves, the log-rank test was used. Gene expression levels were compared by the Student’s t-test. Correlation between the expression of two genes was analyzed by Spearman’s rank correlation coefficient. Statistical analyses were performed by Graphpad prism version 5.0 (GraphPad Software, La Jolla, CA, USA). P-values <0.05 were considered statistically significant.

## Results

### 

#### Decreased expression of CD138 is an indicator of poor prognosis

**Figure 1:**
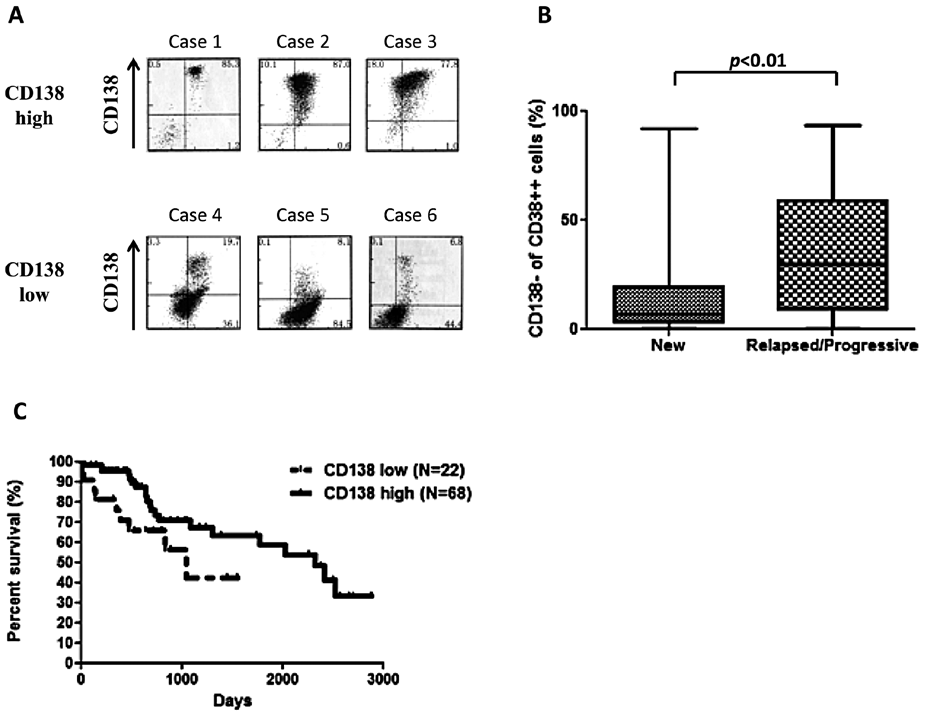
CD138 expression and prognosis. (A) CD138 expression in CD38^++^ multiple myeloma (MM) cells. CD138 expression for 6 representative patients measured by flow cytometry is shown. The upper 3 cases are CD138 high cases. The lower 3 cases are CD138 low cases. (B) Increase in CD138^−^ cells in relapsed or progressive patients. CD138^−^ cells are frequently observed in relapsed or progressive patients (median, 29.8%) compared with newly diagnosed patients (median, 6.6%) (p<0.05, Mann-Whitney U test). (C) CD138 low MM cells are indicators of poor prognosis. In newly diagnosed patients, CD138 low MM patients (n=22) have poor overall survival compared with CD138 high patients (n=68) (p<0.05, log-rank).

Flow cytometric analysis of CD138 expression in MM patients, revealed highly variable expression (Fig. 1A). The number of CD138^−^ cells in the CD38^++^ fraction of the bone marrow from 90 newly diagnosed patients ranged from 0.1% to 91.8% (median, 6.6%), with majority of patients (54%) having less than 10% of CD138^−^ cells. We observed a significant increase in CD138^−^ cells in relapsed or progressive patients (median, 29.8%), compared with the newly diagnosed patients (Fig. 1B. p<0.05, Mann-Whitney U test). CD138 low patients, defined as those patients with greater than 20% CD138^−^ cells, had a worse over overall survival (OS) compared with CD138 high patients (Fig. 1C. p<0.05, log-rank). Survival analysis of patients receiving high-dose chemotherapy followed by autologous stem-cell transplantation also revealed that CD138 low patients had a worse prognosis than that of CD138 high cases (data not shown, p<0.05).

#### Establishment of KYMM-1 and KYMM-2 MM cell lines

**Figure 2:**
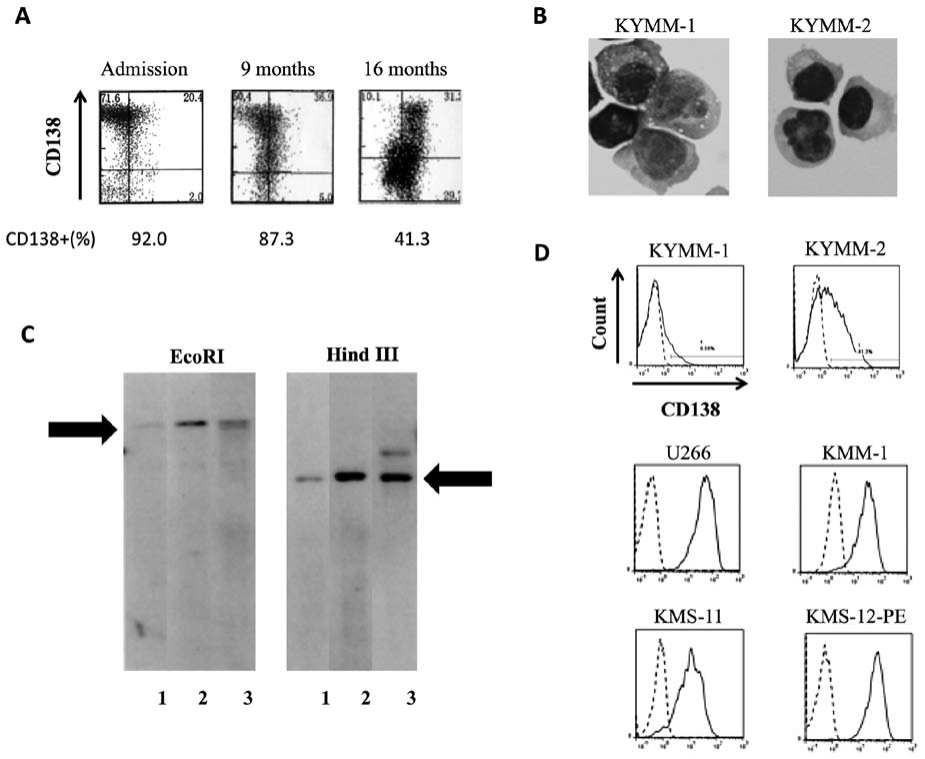
Establishment of KYMM-1 and KYMM-2 MM cell lines. (A) Decreased CD138 expression was observed during the clinical course of the patient from who the KYMM-1 and KYMM-2 cell lines were established. (B) Morphology of KYMM-1 and KYMM-2. (C) Southern blot analysis of the immunoglobulin heavy-chain joining (Ig-JH) region. Lane 1, KYMM-1; lane 2, KYMM-2; lane 3, fresh samples from pleural effusion. DNA was digested by *Eco*RI and *Hin*dIII. Arrows indicate the same rearrangement bands. (D) CD138 expression in KYMM-1, KYMM-2 and other MM cell lines. Differences in CD138 expression were observed in KYMM-1 (8.6%) and KYMM-2 (41.3%) cell lines. Dashed lines, isotype matched control; solid lines, CD138.

KYMM-1 and KYMM-2 cell lines were established from cells obtained from the pleural effusion of a single MM patient, whose CD138 expression decreased during the clinical course (Fig. 2A). KYMM-1 displayed a plasmablastic morphology with cytoplasmic vacuoles, while KYMM-2 exhibited a more mature morphology (Fig. 2B). Southern blot analysis revealed that KYMM-1, KYMM-2 and cells from patient’s pleural effusion shared the same immunoglobulin rearrangement, indicating clonality between these cell lines and primary patient cells (Fig. 2C). DNA profiles of 9 STR-loci and amelogenin were identical among KYMM-1, KYMM-2 and the patient’s pleural effusion cells (data not shown), further confirming that these two cell lines are derived from the same individual. No EB virus genome was detected in the two cell lines.

Despite being established from a single patient, we observed differential expression of CD138 in KYMM-1 (8.6%) and KYMM-2 (41.3%) cell lines (Fig. 2D). In comparison, CD138 was expressed at higher levels in U266, KMM-1, KMS-11 and KMS-12-PE MM cell lines. Flow cytometric analysis of other antigens revealed that both KYMM-1 and KYMM-2 expressed high levels of CD38 and cytoplasmic κ light chain (Cy-κ), while CD45 was highly expressed in KYMM-1 and CD56 was expressed only in KYMM-2 (Table II). CD19 and CD20 were absent in both cell lines.

#### Analysis of gene expression in KYMM-1 and KYMM-2 cells

**Figure 3:**
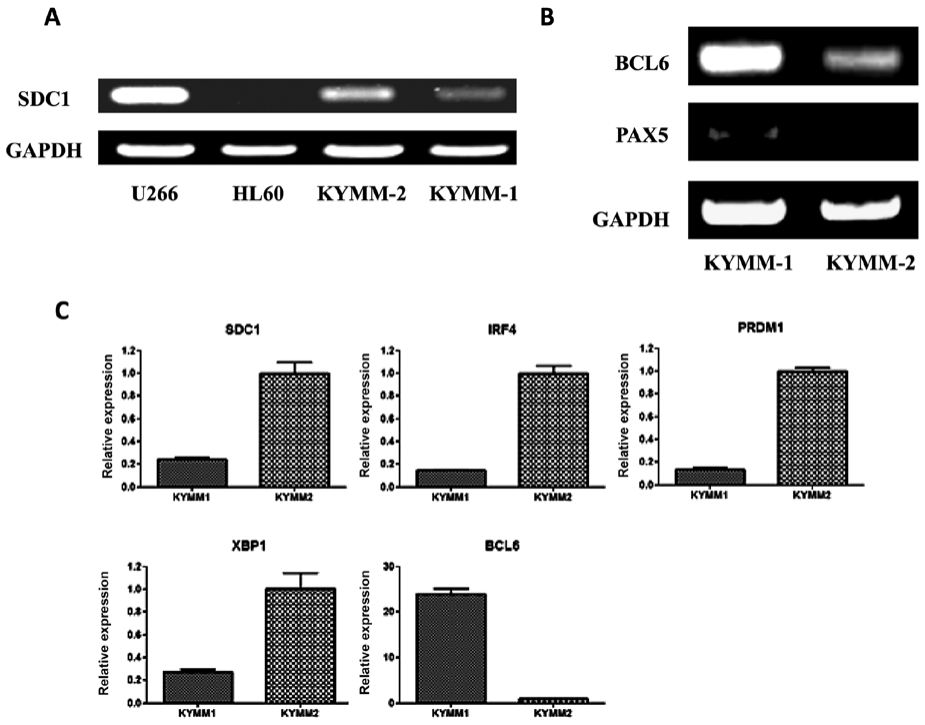
Analysis of gene expression in KYMM-1 and KYMM-2 cell lines. (A) *SDC1* gene expression was analyzed by reverse-transcription polymerase chain reaction (RT-PCR). The U266 MM cell line was used as positive control, while the HL-60 leukemia cell line served as a negative control for *SDC1* expression. *SDC1* was downregulated in KYMM-1. (B) Expression of B-cell transcription factors, *BCL6* and *PAX5*, was analyzed by RT-PCR. Higher expression of *BCL6* was observed in KYMM-1 compared with KYMM-2 cells. *PAX5* was expressed only in KYMM-1. (C) Gene expression was analyzed by real-time RT-PCR. Higher expression of *SDC1*, *IRF4*, *PRDM1* and *XBP1* was observed in KYMM-2 (p <0.01, Student’s t-test), while higher expression of *BCL6* was observed in KYMM-1 cells (p<0.01).

Given the difference in surface antigen expression of CD138 between KYMM-1 and KYMM-2 cells, we examined the expression of *SDC1* mRNA in the two cell lines by RT-PCR (Fig. 3A). In accordance with the observed cell surface expression of CD138, *SDC1* expression was decreased in KYMM-1 compared with KYMM-2. No methylation in the *SDC1* promoter region of KYMM-1 and KYMM-2 was observed by bisulfite sequencing, indicating that the decrease in *SDC1* expression was not related to promoter methylation (data not shown).

Because CD138 expression is highly specific for terminally differentiated plasma cells (1), we hypothesized that KYMM-1 may have a less mature phenotype compared with KYMM-2. To investigate this, we assessed the expression of *BCL6* and *PAX5* transcription factors, which play a role in B-cell differentiation and are downregulated in mature plasma cells (21), by RT-PCR in KYMM-1 and KYMM-2 cells. *BCL6* and *PAX5* expression was clearly detected in KYMM-1 cells, while expression was decreased or absent in KYMM-2 cells (Fig. 3B). Gene expression of *IRF4*, *PRDM1* and *XBP1*, which are also plasma cell specific transcription factors (21), was analyzed by real-time RT-PCR. In accordance with the downregulation of *SDC1* in KYMM-1 cells, *IRF4*, *PRDM1* and *XBP1* levels were also decreased in KYMM-1 compared with KYMM-2 cells (Fig. 3C). Taken together, these results indicate that the CD138 low cell line, KYMM-1 has an immature gene expression profile, compared with KYMM-2.

#### KYMM-1 cells are less sensitive to lenalidomide treatment than KYMM-2

**Figure 4:**
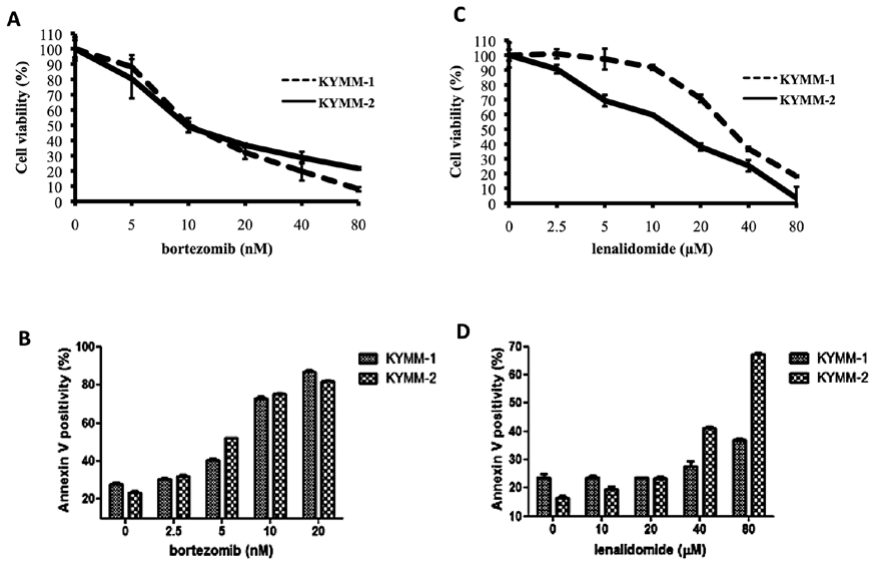
Drug sensitivity of KYMM-1 and KYMM-2 cell lines. (A) KYMM-1 and KYMM-2 were treated with various concentrations of bortezomib and incubated for 24 h. Cell growth was analyzed by WST-8 assay. No difference in sensitivity to bortezomib was observed between the two cell lines. (B) Induction of apoptosis was analyzed by Annexin V staining. KYMM-1 and KYMM-2 were treated with the indicated concentrations of bortezomib for 24 h. The difference in apoptotic cells in the two cell lines following treatment with bortezomib is minimal. (C) KYMM-1 and KYMM-2 were treated with various concentrations of lenalidomide for 72 h and analyzed by WST-8 assay. KYMM-1 was resistant to lenalidomide compared with KYMM-2. (D) Marked increase in Annexin V positive cells was observed in KYMM-2 at 40 *μ*M and 80 *μ*M of lenalidomide compared with KYMM-1.

We next assessed the sensitivity of KYMM-1 and KYMM-2 cells to bortezomib and lenalidomide, to investigate whether their phenotype influenced drug sensitivity. While no significant difference in bortezomib sensitivity was observed between the two cell lines (Fig. 4A and B), KYMM-1 cells were more refractory to lenalidomide compared with KYMM-2 (Fig. 4C). Indeed, we observed a marked increase in Annexin V positive KYMM-2 cells compared with KYMM-1 cells following treatment with 40 and 80 *μ*M of lenalidomide (Fig. 4D). These results indicate that cells expressing decreased levels of CD138 may be resistant to lenalidomide, while bortezomib treatment is equally effective.

#### Correlation of CD138 and IRF4 expression

**Figure 5:**
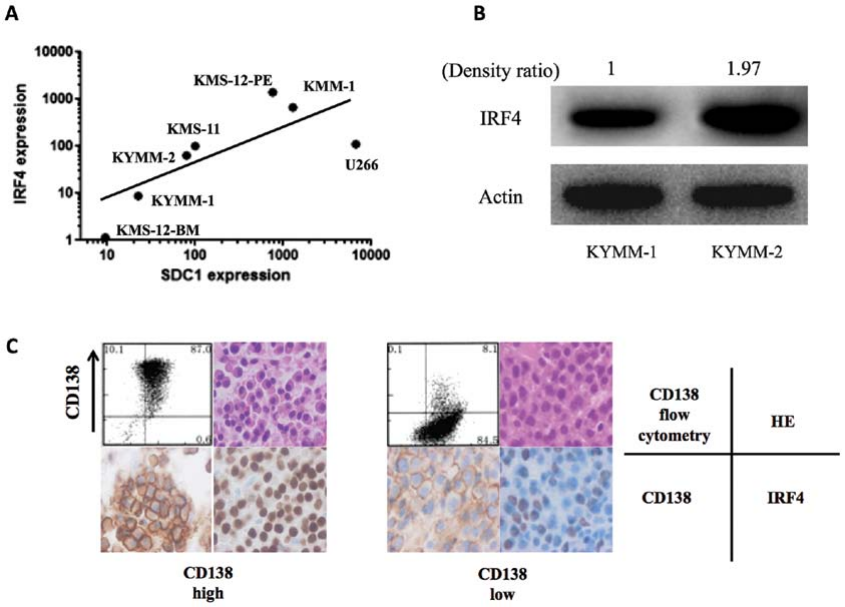
Correlation of CD138 and IRF4 expression. (A) Gene expression of *SDC1* and *IRF4* were analyzed by real-time RT-PCR in seven MM cell lines. A positive correlation was observed between *SDC1* and *IRF4* gene expression (r=0.86, p=0.024, Spearman’s rank correlation coefficient). (B) Western blot analysis of IRF4 in KYMM-1 and KYMM-2 cells. The density ratio was calculated using ImageJ software. Lower IRF4 expression was observed in KYMM-1 compared with KYMM-2 cells. (C) Immunohistochemistry of CD138 and IRF4 in primary MM samples. CD138 and IRF4 were highly expressed in a patient with high CD138 expression by flow cytometry, while CD138 and IRF4 were weakly stained in a CD138 low patient.

Recent studies demonstrate that IRF4 is a key target of lenalidomide, and high IRF4 levels have been correlated with increased lenalidomide sensitivity (22,23). Since the CD138 low MM cell line, KYMM-1, displayed decreased *IRF4* expression and low sensitivity to lenalidomide, we examined the correlation between *SDC1* and *IRF4* expression by real-time RT-PCR in 7 MM cell lines, including KYMM-1 and KYMM-2. We observed a positive correlation between *SDC1* and *IRF4* gene expression (Fig. 5A, r=0.86, p=0.024, Spearman’s rank correlation coefficient). Analysis of IRF4 protein levels by western blot, revealed decreased expression in KYMM-1 compared with KYMM-2 cells (Fig. 5B), which was compatible with the RNA analysis. This tendency was also observed in primary MM cells, since IRF4 downregulation was observed in MM cells with decreased CD138 expression (Fig. 5C). These results indicate that the decrease in CD138 in MM cells accompanies IRF4 downregulation, which may lead to decreased sensitivity to lenalidomide.

#### Fractionation of KYMM-2

**Figure 6:**
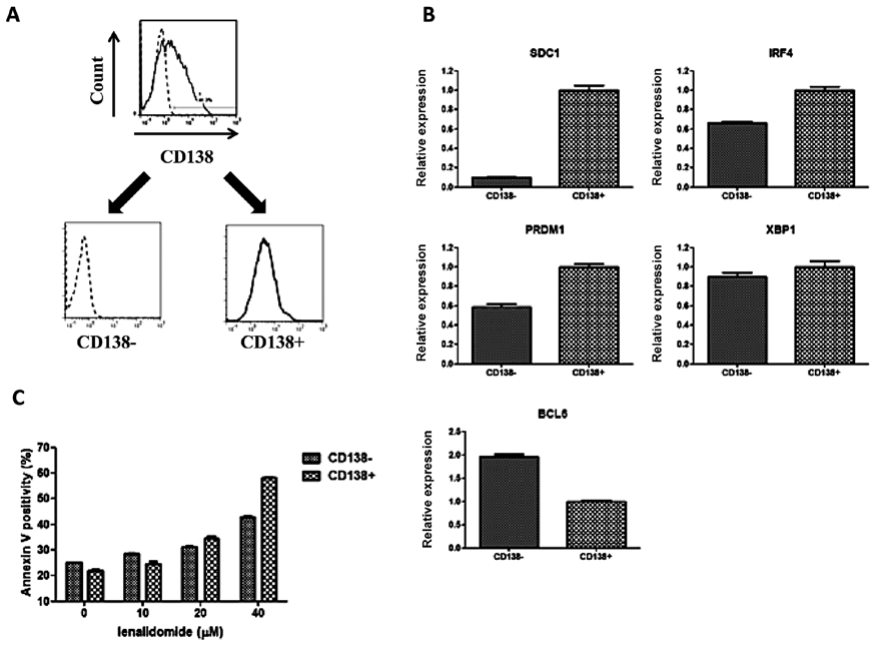
Phenotypic differences in the CD138^+^ and CD138^−^ fraction of KYMM-2 cells. (A) KYMM-2 was fractionated into CD138^+^ and CD138^−^ populations using CD138 magnetic beads. The purity of each fraction was approximately 90%. (B) Gene expression between the two fractions was analyzed by real-time RT-PCR. Higher expression of *SDC1*, *IRF4* and *PRDM1* was observed in CD138^+^ fraction (p<0.01, Student’s t-test), while higher expression of *BCL6* observed in CD138^−^ fraction (p<0.01). (C) An increase in Annexin V positive cells was observed at 40 *μ*M of lenalidomide in the CD138^+^ fraction compared with CD138^−^ fraction.

To further analyze the phenotype related to CD138 expression, we sorted KYMM-2 cells into CD138^+^ and CD138^−^ fractions using CD138 magnetic beads (Fig. 6A). As observed in our previous comparison, we also found that both *IRF4* and *PRDM1* gene expression was elevated in the CD138^+^ fraction, while *BCL6* expression was highest in the CD138^−^ fraction (Fig. 6B). Furthermore, an increase in Annexin V positive cells was observed at 40 *μ*M of lenalidomide in the CD138^+^ fraction compared with CD138^−^ fraction, suggesting reduced sensitivity of CD138^−^ cells to lenalidomide (Fig. 6C). These data demonstrate that CD138^−^ and CD138^+^ fractions obtained from KYMM-2 have similar phenotypes as identified in KYMM-1 and KYMM-2, although the differences were not as large as those observed between KYMM-1 and KYMM-2.

## Discussion

The aim of this study was to determine the characteristics and the clinical significance of CD138^−^ MM cells. In the present study, variability in CD138 expression in MM cells was observed as previously reported (7,8). Importantly, we observed an increase in CD138^−^ cells in relapsed or progressive patients (median, 29.8%) compared with newly diagnosed patients (median, 6.6%). Therefore, population of refractory disease status should influence the outcome of CD138 expression analysis. For example, the higher frequency of CD138^−^ MM cells reported by Reid *et al*(8) may be attributed to the significant number of refractory cases in this patient cohort.

Previous studies have reported that the mean fluorescence intensity (MFI) of CD138 significantly decreases in MM cells compared with normal plasma cells (24), suggesting that CD138^+^ MM cells are more closely related to normal plasma cells compared with their CD138^−^ counterparts. This hypothesis may be compatible with our results focusing on untreated cases, which showed that CD138 low patients had poor prognosis compared with CD138 high patients. Interestingly, even patients receiving high-dose chemotherapy showed similar results (data not shown). To our knowledge, this is the first report demonstrating a correlation between CD138 expression and MM prognosis. Since our study is a retrospective analysis in a heterogeneous group of patients, the data should be investigated further in a prospective randomized study. However, CD138 low cells exist in a subset of MM patients and should be evaluated carefully, since CD138 expression has previously been considered as a hallmark of MM cells (5). Identification of MM cells using CD138 expression as a sole identifying factor, may fail to identify CD138^−^ MM cells.

We have established two MM cell lines, KYMM-1 and KYMM-2, from a patient expressing declining levels of CD138. To our knowledge, the KYMM-1 cell line is one of the lowest CD138-expressing MM cell lines, compared with the previously established cell lines (25). While KYMM-1 expresses low levels of CD138, it is still considered a myeloma cell line, since it expresses high levels of CD38 and the cytoplasmic kappa light chain, and lacks CD19 expression and the EB virus genome. CD45, which is also highly expressed on KYMM-1 cells, is considered to be associated with a more primitive plasma cell phenotype (26), indicating that KYMM-1 is less mature than KYMM-2. Furthermore, our analysis of transcription factors revealed preferential expression of genes expressed at the immature B-cell stage compared with terminally differentiated plasma cells, such as *BCL6* and *PAX5*, and lower expression of genes associated with mature plasma cells, including *IRF4*, *PRDM1* and *XBP1* in KYMM-1 compared with KYMM-2. A similar pattern of gene expression was also identified in the CD138^−^ fraction of KYMM-2 cells. These findings are not surprising, since previous reports have shown that B-cell transcription factors such as BCL6 and PAX5, are expressed in a subset of MM cells (5,27,28). Another report showed that the expression of SDC1 in MM cells decreased following adhesion to stromal cells, which was accompanied by an increase in the ratio of BCL6 to PRDM1 expression (29). This indicates that CD138 is not always overexpressed in MM cells, and is dynamically regulated in accordance with transcription factors related to B-cell maturation. In this study, we hypothesize that CD138 down-regulation may require immature transcription status.

CD138 negative cells have recently been proposed as myeloma stem cells. Based on previous studies by Matsui *et al*, which reported that MM stem cells express CD138^−^ CD20^+^ CD27^+^(30), we assessed the surface expression of CD20 and CD27 in KYMM-1 and KYMM-2 cell lines. This analysis revealed that neither KYMM-1 nor KYMM-2 expressed CD20 or CD27. Analysis of ALDH activity, which is a characteristic of MM stem cells (30,31), revealed no difference between KYMM-1 and KYMM-2 cells (data not shown). Thus, while KYMM-1 cells displayed an immature gene expression profile, there is no evidence thus far that it exhibits MM stem cell-like features.

In this study, we found that low expression of CD138 was an adverse prognostic factor, even in patients treated with high-dose chemotherapy. Interestingly, while no difference in sensitivity to bortezomib was observed between the two MM cell lines, KYMM-1 was more resistant to lenalidomide compared with KYMM-2. This tendency was also observed in a subset of KYMM-2 cells with low CD138 expression. Recent reports show that high IRF4 expression correlates with increased lenalidomide sensitivity (22,23). Our analysis revealed that downregulation of IRF4 in KYMM-1 may provide a mechanism for the observed lower sensitivity of KYMM-1 to lenalidomide. Our finding showing the correlation between *IRF4* and *SDC1* expression in MM cell lines may also explain the poor response of CD138 low patients to lenalidomide, which may contribute to poor prognosis, although only a small number of heterogeneously treated cases were analyzed. Additional detailed, prospective investigations in a homogenously treated cohort are required to draw a definitive conclusion.

Taken together, our observations suggest that low CD138 expression is associated with i) poor prognosis, ii) immature phenotype and iii) refractoriness to lenalidomide. The observed, distinct characteristics of CD138 low MM cells, suggest that this should be recognized as a new clinical entity. Establishment of a treatment strategy for patients with CD138 low MM cells is necessary to improve their poor outcome.

## Figures and Tables

**Table I:** Patient characteristics.

	CD138 high	CD138 low
Median CD138^−^ (%) (Range)	4.7 (0.1–18.6)	30(20.6–91.8)
Median age (Range)	65 (44–85)	65(44–82)
Gender		
Male	37	13
Female	31	9
Total	68	22

CD138^−^ (%), CD138 negative cells (%) in CD38^++^ fraction.

**Table II:** Surface antigen expression of KYMM-1 and KYMM-2.

	KYMM-1	KYMM-2
CD19	0	0
CD20	0	0
CD27	0	0
CD34	0	0
CD38	92.7	98.2
CD44	99.5	98.3
CD45	92.1	35.9
CD54	93.1	98.4
CD56	0	43
CD138	8.6	41.3
Cy-κ	95.7	97.2

The numbers indicate % expression. Cy-κ, cytoplasmic-κ light chain.

## References

[1] Calame KL (2001). Plasma cells: finding new light at the end of B cell development. Nat Immunol.

[2] Dhodapkar MV, Abe E, Theus A (1998). Syndecan-1 is a multi-functional regulator of myeloma pathobiology: control of tumor cell survival, growth, and bone cell differentiation. Blood.

[3] Ridley RC, Xiao H, Hata H, Woodliff J, Epstein J, Sanderson RD (1993). Expression of syndecan regulates human myeloma plasma cell adhesion to type I collagen. Blood.

[4] Wijdenes J, Vooijs WC, Clement C (1996). A plasmocyte selective monoclonal antibody (B-B4) recognizes syndecan-1. Br J Haematol.

[5] Zhan F, Huang Y, Colla S (2006). The molecular classification of multiple myeloma. Blood.

[6] Ikeda H, Hideshima T, Fulciniti M (2009). The monoclonal antibody nBT062 conjugated to cytotoxic Maytansinoids has selective cytotoxicity against CD138-positive multiple myeloma cells in vitro and in vivo. Clin Cancer Res.

[7] Witzig TE, Kimlinger T, Stenson M, Therneau T (1998). Syndecan-1 expression on malignant cells from the blood and marrow of patients with plasma cell proliferative disorders and B-cell chronic lymphocytic leukemia. Leuk Lymphoma.

[8] Reid S, Yang S, Brown R (2010). Characterisation and relevance of CD138-negative plasma cells in plasma cell myeloma. Int J Lab Hematol.

[9] Matsui W, Huff CA, Wang Q (2004). Characterization of clonogenic multiple myeloma cells. Blood.

[10] Harada H, Kawano MM, Huang N (1993). Phenotypic difference of normal plasma cells from mature myeloma cells. Blood.

[11] Ohmori M, Nagai M, Fujita M (1998). A novel mature B-cell line (DOBIL-6) producing both parathyroid hormone-related protein and interleukin-6 from a myeloma patient presenting with hypercalcaemia. Br J Haematol.

[12] Togawa A, Inoue N, Miyamoto K, Hyodo H, Namba M (1982). Establishment and characterization of a human myeloma cell line (KMM-1). Int J Cancer.

[13] Namba M, Ohtsuki T, Mori M (1989). Establishment of five human myeloma cell lines. In Vitro Cell Dev Biol.

[14] Ohtsuki T, Yawata Y, Wada H, Sugihara T, Mori M, Namba M (1989). Two human myeloma cell lines, amylase-producing KMS-12-PE and amylase-non-producing KMS-12-BM, were established from a patient, having the same chromosome marker, t(11;14)(q13;q32). Br J Haematol.

[15] Nilsson K, Bennich H, Johansson SG, Ponten J (1970). Established immunoglobulin producing myeloma (IgE) and lymphoblastoid (IgG) cell lines from an IgE myeloma patient. Clin Exp Immunol.

[16] Birnie GD (1988). The HL60 cell line: a model system for studying human myeloid cell differentiation. Br J Cancer.

[17] Mahmoud MS, Huang N, Nobuyoshi M, Lisukov IA, Tanaka H, Kawano MM (1996). Altered expression of Pax-5 gene in human myeloma cells. Blood.

[18] Tatetsu H, Ueno S, Hata H (2007). Down-regulation of PU.1 by methylation of distal regulatory elements and the promoter is required for myeloma cell growth. Cancer Res.

[19] Livak KJ, Schmittgen TD (2001). Analysis of relative gene expression data using real-time quantitative PCR and the 2(−Delta Delta C(T)) method. Methods.

[20] Matsuno N, Hoshino K, Nanri T (2005). p15 mRNA expression detected by real-time quantitative reverse transcriptase-polymerase chain reaction correlates with the methylation density of the gene in adult acute leukemia. Leuk Res.

[21] Shapiro-Shelef M, Calame K (2005). Regulation of plasma-cell development. Nat Rev Immunol.

[22] Lopez-Girona A, Heintel D, Zhang LH (2011). Lenalidomide downregulates the cell survival factor, interferon regulatory factor-4, providing a potential mechanistic link for predicting response. Br J Haematol.

[23] Li S, Pal R, Monaghan SA (2009). IMiD immunomodulatory compounds block C/EBP{beta} translation through eIF4E down-regulation resulting in inhibition of MM. Blood.

[24] Peceliunas V, Janiulioniene A, Matuzeviciene R, Griskevicius L (2011). Six color flow cytometry detects plasma cells expressing aberrant immunophenotype in bone marrow of healthy donors. Cytometry B Clin Cytom.

[25] Bataille R, Jego G, Robillard N (2006). The phenotype of normal, reactive and malignant plasma cells. Identification of ‘many and multiple myelomas’ and of new targets for myeloma therapy. Haematologica.

[26] Pope B, Brown RD, Gibson J, Yuen E, Joshua D (2000). B7-2-positive myeloma: incidence, clinical characteristics, prognostic significance, and implications for tumor immunotherapy. Blood.

[27] Lin P, Mahdavy M, Zhan F, Zhang HZ, Katz RL, Shaughnessy JD (2004). Expression of PAX5 in CD20-positive multiple myeloma assessed by immunohistochemistry and oligonucleotide microarray. Mod Pathol.

[28] Hideshima T, Mitsiades C, Ikeda H (2010). A proto-oncogene BCL6 is up-regulated in the bone marrow microenvironment in multiple myeloma cells. Blood.

[29] Fuhler GM, Baanstra M, Chesik D (2010). Bone marrow stromal cell interaction reduces syndecan-1 expression and induces kinomic changes in myeloma cells. Exp Cell Res.

[30] Matsui W, Wang Q, Barber JP (2008). Clonogenic multiple myeloma progenitors, stem cell properties, and drug resistance. Cancer Res.

[31] Brennan SK, Wang Q, Tressler R (2010). Telomerase inhibition targets clonogenic multiple myeloma cells through telomere length-dependent and independent mechanisms. PLoS One.

